# Tumor spread through air spaces in lung cancer: prospective analysis of the accuracy of intraoperative frozen section examination

**DOI:** 10.36416/1806-3756/e20240165

**Published:** 2024-09-16

**Authors:** Germano Luciano de Almeida, Bruno Maineri Pinto, Vitor Maineri Pinto, Aline Caldart Tregnago, Renata Fragomeni Almeida, Darcy Ribeiro Pinto

**Affiliations:** 1. Hospital Geral de Caxias do Sul, Universidade de Caxias do Sul, Caxias do Sul (RS) Brasil.

**Keywords:** Neoplasm invasiveness, Frozen sections, Lung neoplasms/pathology, Lung neoplasms/surgery

## Abstract

**Objective:**

To establish the accuracy of frozen section examination in identifying tumor spread through air spaces (STAS), as well as to propose a reproducible technical methodology for frozen section analysis. We also aim to propose a method to be incorporated into the decision making about the need for conversion to lobectomy during sublobar resection.

**Methods:**

This was a nonrandomized prospective study of 38 patients with lung cancer who underwent surgical resection. The findings regarding STAS in the frozen section were compared with the definitive histopathological study of paraffin-embedded sections. We calculated a confusion matrix to obtain the positive predictive value (PPV), negative predictive value (NPV), sensitivity, specificity and accuracy.

**Results:**

The intraoperative frozen section analysis identified 7 STAS-positive cases that were also positive in the histopathological examination, as well as 3 STAS-negative cases that were positive in the in the histopathological examination. Therefore, frozen section analysis was determined to have a sensitivity of 70%, specificity of 100%, PPV of 100%, NPV of 90.3%, and accuracy of 92% for identifying STAS.

**Conclusions:**

Frozen section analysis is capable of identifying STAS during resection in patients with lung cancer. The PPV, NPV, sensitivity, and specificity showed that the technique proposed could be incorporated at other centers and would allow advances directly linked to prognosis. In addition, given the high accuracy of the technique, it could inform intraoperative decisions regarding sublobar versus lobar resection.

## INTRODUCTION

The concept of tumor spread through air spaces (STAS) is defined by the World Health Organization (WHO) Classification of Tumors as “micropapillary clusters, solid nests, or single cells spreading within air spaces beyond the edge of the main tumor.”^1^ Although the concept of STAS varies across studies, the WHO concept is the most widely accepted and disseminated in the literature. Although Kodama et al.^2^ first identified STAS in 1980, a debate began in 2023 when Onozato et al.^3^ identified tumor islands as a factor for worse five-year disease-free survival in early-stage lung adenocarcinomas: 44.6% in patients with tumor islands and 74.4% in those without. In 2015, Kadota et al.^4^ introduced the concept of STAS to the world by reporting a cumulative five-year recurrence rate of 42.6% in STAS-positive patients who underwent sublobar resection, compared with 12.7% in those undergoing lobectomy. In that same year, Warth et al.^5^ evaluated a cohort of 569 patients with lung adenocarcinoma and found that overall survival and five-year disease-free survival were worse among those with STAS, as well as establishing important definitions regarding this pathological entity. Subsequent studies, conducted between 2015 and 2018, presented similar results and pointed out new findings.^6^ ^-^ ^11^ All of these studies identified STAS in the postoperative period, which led to a need to detect this important risk factor in the intraoperative period in order to be able to convert sublobar resections into lobectomies. In 2018, Walts et al.^12^ were pioneers in the study of intraoperative frozen pathology for this purpose, despite the fact that they obtained negative results.

A meta-analysis conducted by Chen et al.,^13^ involving a collective total of 3,754 patients in 14 studies, suggested that the presence of STAS was associated with worse recurrence-free survival and overall survival in non-small cell lung cancer (NSCLC). Subgroup analysis by histological type indicated that the presence of STAS was significantly associated with worse recurrence-free survival after resection of lung adenocarcinoma, lung squamous cell carcinoma, or pleomorphic lung carcinoma. It was also related to shorter overall and recurrence-free survival, regardless of tumor stage.^13^ ^,^ ^14^ The presence of STAS was also more frequently observed in advanced-stage NSCLC, exhibiting prognostic value in all pathological stages. It is also an unfavorable prognostic factor in pulmonary metastases from colorectal cancer, although its role is not yet fully defined.^14^ Therefore, given the high risk of locoregional recurrence in patients with STAS, the treatment standard for these patients may be lobectomy instead of sublobar resection, whether the patient meets the criteria for segmentectomy or not.^13^ ^,^ ^15^

Given the data in the literature demonstrating the negative impact that STAS has on patient survival, the major dilemma is regarding the timing of diagnosis. A finding of STAS in the postoperative analysis of sublobar resection poses a pertinent question: should we proceed with lobectomy, necessitating a return to the operating room, or simply observe the progression? Obviously, if we could decide intraoperatively, through frozen section pathology, this dilemma would be resolved. Intraoperative frozen section is a well-known technique widely used by pathologists. However, there are some obstacles to its use in the setting of lung cancer, especially because of the lack of a defined protocol to serve as a basis. Another important factor is that STAS is also often difficult to distinguish from intra-alveolar macrophages, a distinction that must be made on the basis of the nucleus-cytoplasm ratio and degree of nuclear atypia.^15^ Therefore, our aim was to establish the accuracy of intraoperative frozen section pathology in identifying STAS, as well as to propose a technical methodology for frozen section pathology analysis that can be reproduced. In addition, on the basis of our findings, we aim to propose a method to be incorporated into the decision-making process regarding the need for lobectomy in indications for sublobar resection.

## METHODS

This was a prospective, nonrandomized study in which patients with a preoperative or intraoperative diagnosis of lung cancer underwent diagnostic or therapeutic resection. During the procedure, the surgical specimen was analyzed through frozen section pathology. If a diagnosis of STAS was confirmed, the surgical team, along with the pathologist, indicated conversion from sublobar resection to lobectomy. Categorically, conversion was not indicated only in cases of metastasis from other sites, of inadequate pulmonary reserve, and of advanced tumors. Subsequent to the procedure, the surgical specimen was sent for definitive histopathological analysis of paraffin-embedded sections, after which the details of the case were entered into a database and the results were compared. The study was approved by the Scientific and Editorial Committee of the *Hospital Geral de Caxias do Sul*, operated by the University of Caxias do Sul, in the city of Caxias do Sul, Brazil. All participating patients gave written informed consent.

We identified 44 patients who underwent lung resection for diagnosis or definitive treatment between April 2020 and October 2022 in the Thoracic Surgery Department of the *Hospital Geral de Caxias do Sul*. It is worth noting that the COVID-19 pandemic imposed certain difficulties during that interval, especially at high-complexity referral hospitals like ours. Of the 44 patients initially selected, 6 were excluded: 1 because the result was inconclusive; and 5 because the frozen section was not screened for STAS by the pathologist who was part of the study, thus maintaining the prospective nature of the study. This resulted in a total of 38 patients: 28 who were negative for STAS (in 22 sublobar resections and 6 lobectomies); and 10 who were positive for STAS (in 6 sublobar resections and 4 lobectomies). We emphasize that, regardless of the STAS status, tumors for which lobectomy was indicated were subjected to such.

### Histological analysis

The histological analysis compared the results of the STAS investigation in frozen sections with the definitive histopathological study of paraffin-embedded sections. Using a standardized histological technique based on the previous experience of the team and the international literature, two pathologists interpreted the intraoperative findings. However, in each case, the intraoperative finding and the histopathological result were both evaluated by the same pathologist thereby avoiding case overlap.

**Figure 1:**
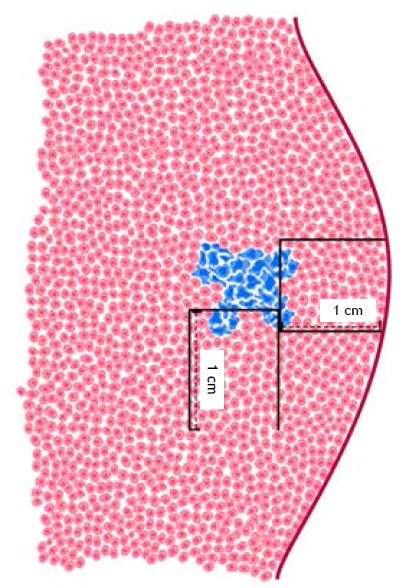
Illustration of material sampling for frozen section examination.

**Figure 2:**
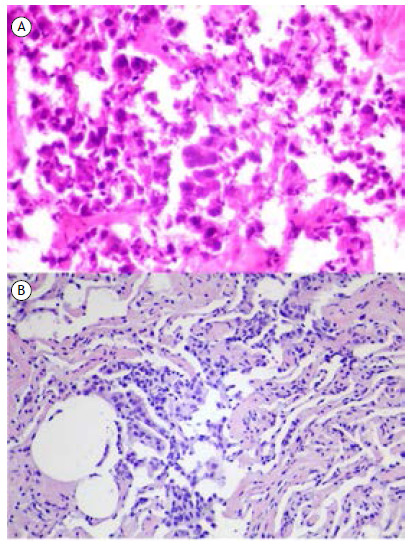
Example case demonstrating tumor spread through air spaces in the intraoperative frozen section (A) and in the paraffin block (B).

For the intraoperative frozen section examination, two randomly selected sections from different areas of the parenchyma immediately adjacent to the lesion were sampled. These sections encompassed a small portion of the lesion, the transition to the adjacent parenchyma, and the adjacent parenchyma up to 1.0 cm away when possible (Figure 1). The distance of 1.0 cm was chosen because STAS is evaluated at the tumor margins, despite the absence of a specific measurement for this evaluation in the current literature. Histological sections of 4-5 µm in thickness were cut in a cryostat and stained with routine hematoxylin and eosin. To increase the sensitivity of the intraoperative assessment without prolonging the examination time, only two sections were selected; ideally, the entire tumor margin should be evaluated, but this is unfeasible due to time constraints. The result was communicated to the surgeon and documented in writing. Figure 2 illustrates the presence of STAS in one of our cases.

For the histopathological examination, the same histological sections used in the intraoperative frozen section examination were embedded in paraffin to prepare histological slides, cut to a thickness of 3 µm, and stained with hematoxylin and eosin. Additional sections were included to represent the tumor, following the sampling recommendations and guidelines for histopathological reports established by the College of American Pathologists.^16^ The inclusion of additional paraffin-embedded sections may facilitate the identification of STAS in areas not sampled during the frozen section analysis.

### Statistical analysis

The database was created with information collected from the electronic medical records of the selected patients. The categorical (dichotomous) diagnosis of STAS (i.e., STAS positivity and STAS negativity) made by the evaluating pathologist from the frozen sections was compared with the results of the evaluation of paraffin-embedded sections by the same pathologist. After finalizing the counts related to the categorizations obtained with the two techniques, we calculated a confusion matrix to determine the positive predictive value (PPV), negative predictive value (NPV), sensitivity, and specificity of frozen section analysis. Those values were used to determine the accuracy of the method. In addition, a ROC curve was constructed to compare intraoperative frozen section examination with histopathological examination, by determining the AUC. Variables investigated for potential associations with STAS positivity were defined on the basis of previous studies, and chi-square tests were applied to qualitative variables, whereas Student’s t-tests were used for quantitative variables. Means and medians were calculated for continuous variables, and standard deviations were computed for subsamples. Categorical variables are presented as counts and percentages. Statistical analysis was performed with the IBM SPSS Statistics software package, version 27.0 (IBM Corp., Armonk, NY, USA).

## RESULTS

### Comparison between frozen section analysis and definitive histopathological diagnosis

The intraoperative frozen section examination identified 7 patients as STAS positive and 31 as STAS negative, whereas the definitive histopathological examination identified 10 and 28 patients, respectively, as such (Table 1). The frozen section examination identified 7 cases (18.4%) as STAS-positive cases and 3 as STAS-negative cases, those 3 being identified as STAS-positive cases in the histopathological examination of the paraffin-embedded sections, resulting in a sensitivity of 70%, specificity of 100%, PPV of 100%, and NPV of 90.3%. These data are presented in a confusion matrix in Table 1. Five previous studies have also compared intraoperative frozen section examination with definitive paraffin-based histopathology,^12^ ^,^ ^17^ ^-^ ^20^ as detailed in Table 2.

**Table 1:** Confusion matrix of the results of the intraoperative frozen section examination and the histopathological analysis.

Frozen section	Definitive histopathology		Total
	STAS-positive	STAS-negative	
STAS-positive	7	0	7
STAS-negative	3	28	31
Total	10	28	38

STAS: spread through air spaces.

**Table 2:** Comparison among previous studies that compared intraoperative frozen section examination and definitive histopathological analysis of paraffin-embedded sections.

Reference	Year	N	AUC	Sensitivity	Specificity	Accuracy
Walts et al.^12^	2018	48	-	48%	100%	-
Eguchi et al.^17^	2019	48	-	71%	92%	-
Villalba et al.^18^	2021	100	0.67	44%	91%	71%
Zhou et al.^19^	2021	163	-	55%	80%	74%
Ding et al.^20^	2023	294	-	55%	85%	74%
This study	2024	38	0.85	70%	100%	92%

**Figure 3:**
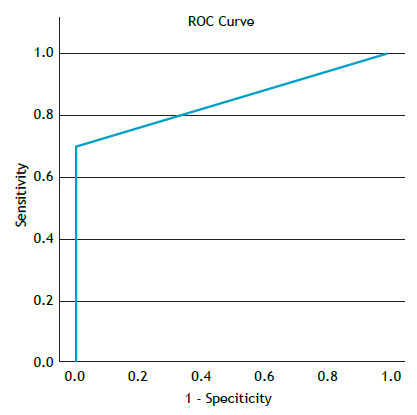
ROC curve of tumor spread through air spaces in the frozen section examination.

The frozen section examination identified the majority of positive cases, and all negative cases in the histopathological examination had the same result in the intraoperative examination, translating to a test accuracy of 92% and an AUC of 0.850 (Figure 3).

### Characteristics of the STAS-positive and STAS-negative cases in the definitive histopathology

Our study sample comprised 38 patients, with a mean age at the time of the surgical procedure of 69.66 ± 12.13 years. Of those 38 patients, 28 (73.7%) had a negative STAS result on histopathological examination and 10 had a positive STAS result (26.3%), with mean ages of 70.39 years (median, 73 years) and 67.6 years (median, 66.5 years), respectively. Sublobar resection was performed in 28 cases (73.7%), and lobectomy was performed in 10 (26.3%).

Among the 28 patients who were negative for STAS, the median age was 73 years (mean, 70.39 ± 10.6 years), compared with a median of 66.5 years (mean, 67.6 ± 16.2 years) among the 10 positive cases (p = 0.041). The mean tumor size was 1.81 ± 1.22 cm in the STAS-negative cases and 2.07 ± 1.02 cm in the STAS-positive cases (p = 0.697). In the STAS-negative subgroup, 79% of the surgical procedures were sublobar resections and 21% were lobectomies, whereas, in the STAS-positive subgroup, 60% were wedge resections or segmentectomies and 40% were lobectomies (p = 0.264). The proportion of cases with Lung CT Screening Reporting and Data System category 4X findings was similar between the two subgroups (p = 0.436). In the STAS-positive subgroup, the predominant tumor type (in 30%) was adenocarcinoma with an acinar pattern, 50% of the tumors showed vascular invasion, 40% of the patients were nonsmokers, and 60% of the patients had a history of cancer other than lung cancer (p = 0.71). There were no significant differences between the two subgroups in terms of tumor necrosis (p = 0.757), involvement of visceral pleura (p = 0.781), or bronchial invasion (p = 0.951), unlike angiolymphatic invasion, which was observed in 32% of the patients in the STAS-negative subgroup and in 50% of those in the STAS-positive subgroup (p = 0.328). The proportion of never-smokers was 40% in the STAS-positive subgroup, compared with 25% in the STAS-negative subgroup (p = 0.16). In addition, 60% of the patients in the STAS-positive subgroup had a history of neoplasia at a site other than the lungs (Table 3), with the vast majority being colorectal (p = 0.475). It should be noted that these findings are not statistically relevant given the small sample size, intergroup heterogeneity, and convenience sampling. However, the chi-square tests for frozen and histopathological sections coincide and are consistent with findings in the international literature. The same was true for the Student’s t-tests for patient age and tumor size (see the supplementary material).

**Table 3:** Demographic and clinical characteristics of the sample (N = 38), by histopathological subgroup.

Characteristic	STAS-negative	STAS-positive	p-value
	(n = 28)	(n = 10)	
Age (years), median (mean ± SD)	73 (70.39 ± 10.6)	66 (67.6 ± 16.2)	0.041
Sex, n (%)			
Female	16 (57.1)	6 (60.0)	0.879
Male	12 (42.8)	4 (40.0)	
Tumor size (cm), mean ± SD	1.81 ± 1.22	2.07 ± 1.02	0.697
Type of resection, n (%)			
Sublobar	22 (78.6)	6 (60.0)	0.264
Lobar	6 (21.4)	4 (40.0)	
Lung-RADS category, n (%)			
4A	4 (14.2)	1 (10.0)	0.436
4B	6 (21.4)	3 (30.0)	
4X	18 (64.4)	6 (60.0)	
Histology, n (%)			
Colorectal metastasis	5 (17.8)	1 (10.0)	0.71
Solid adenocarcinoma	5 (17.8)	1 (10.0)	
Lepidic adenocarcinoma	5 (17.8)	1 (10.0)	
Acinar adenocarcinoma	6 (21.4)	3 (30.0)	
Mucinous adenocarcinoma	3 (10.7)	2 (20.0)	
Papillary adenocarcinoma	1 (3.6)	0	
Micropapillary adenocarcinoma	0	2 (20.0)	
Poorly differentiated adenocarcinoma	1 (3.6)	0	
Carcinosarcoma	1 (3.6)	0	
Atypical carcinoid	1 (3.6)	0	
Tumor necrosis, n (%)			
Yes	7 (25.0)	2 (20.0)	0.757
No	21 (75.0)	8 (80.0)	
Involvement of visceral pleura, n (%)			
Yes	2 (7.1)	1 (10.0)	0.781
No	26 (92.8)	9 (90.0)	
Bronchial invasion, n (%)			
Yes	3 (10.7)	1 (10.0)	0.951
No	25 (89.3)	9 (90.0)	
Angiolymphatic invasion, n (%)			
Yes	9 (32.1)	5 (50.0)	0.328
No	19 (67.8)	5 (50.0)	
Smoking status, n (%)			
Never smoker	7 (25.0)	4 (40.0)	0.16
Current smoker	11 (39.3)	1 (10.0)	
Former smoker (≥ 20 pack-year history)	10 (35.7)	5 (50.0)	
History of lung cancer, n (%)			
Yes	6 (21.4)	1 (10.0)	0.437
No	22 (78.6)	9 (90.0)	
History of other cancer, n (%)			
Yes	13 (46.4)	6 (60.0)	0.475
No	15 (53.6)	4 (40.0)	

STAS: spread through air spaces; and Lung-RADS: (American College of Radiology) Lung CT Screening Reporting and Data System.

## DISCUSSION

In recent decades, thoracic surgery has progressively moved toward minimally invasive procedures and non-radical resection. The discussion on sublobar resection necessarily involves identifying STAS in the preoperative or intraoperative assessment. The method proposed for this purpose is intraoperative frozen section examination, for which there are discrepancies in the literature related to its use. In the present study, with technical standardization, STAS was recognized in the frozen section examination, which showed high (70%) sensitivity and robust (100%) specificity, resulting in an accuracy of 92%. These findings are significant and pave the way for intraoperative identification of STAS, with important prognostic value, in pulmonary resection, especially sublobar resection, in Brazil.

Walts et al.^12^ were the first to compare intraoperative frozen section examination with definitive paraffin-based histopathology, in 2018. Those authors evaluated 48 patients with stage T1 or T2 tumors, 46 of whom were positive for STAS. They found that intraoperative frozen section pathology had a sensitivity of 48%, specificity of 100%, PPV of 100%, and NPV of 8%. However, the authors did not clearly describe a standardized protocol for the frozen section pathology technique and obtained limited samples of normal lung parenchyma adjacent to the lesion.^12^ The following year, Eguchi et al.^17^ analyzed a sample of 48 T1N0 adenocarcinomas, evaluated by 5 different pathologists, who achieved a sensitivity of 71%, specificity of 92%, and 75% agreement among them. The authors did not describe the frozen section methodology, using only the criterion of the resected non-neoplastic adjacent parenchyma being at least one third the size of the main tumor.^17^ Villalba et al.,^18^ in a sample of 100 patients analyzed by 3 pathologists, obtained an AUC of 0.67, sensitivity of 44%, specificity of 91%, accuracy of 71%, PPV of 79.2%, and NPV of 68.4%. They also reported moderate interobserver agreement among pathologists who made the analyses and intraobserver agreement (because the slides were analyzed more than once) ranging from 77% to 85%. Again, no frozen section methodology was described.^18^ In a 2021 study, Zhou et al.^19^ evaluated 163 stage I adenocarcinomas and were the first to clearly describe the methodology of intraoperative frozen section examination. Those authors found the technique to have a sensitivity of 55%, specificity of 80%, accuracy of 74%, PPV of 48%, and NPV of 85%. It is noteworthy that they described various artifacts as exclusion factors for the diagnosis of STAS, which could explain the high false-positive rate.^19^ In a 2023 study, Ding et al.^20^ retrospectively analyzed frozen section examination in a sample of 294 patients with NSCLC, demonstrating that it had an accuracy of 74.14%, sensitivity of 55.14%, and specificity of 85.02%, with discordance between the cases with a consolidation-to-tumor ratio (CTR) ≤ 0.5 and those with a CTR > 0.5, the results being more satisfactory in the latter. Thus, they concluded that intraoperative frozen section examination is applicable in cases of NSCLC with a CTR > 0.5.^20^ Bearing these data in mind, we observe that all previous studies have evaluated frozen section examination retrospectively, with ours being the first prospective study of the topic.

A study conducted by Metovic et al.^21^ aimed to investigate whether gross specimen handling procedures influence the rate of STAS detection in cases of lung cancer. The study involved a prospective analysis of 51 surgical lung specimens, encompassing various histological types. Each specimen was meticulously handled, with fresh tissue sections cut using a new blade for each incision, followed by separate processing after formalin fixation. The authors identified STAS in 64.7% of the cases, predominantly as clustered formations (in 87.9%). It is noteworthy that they found no significant difference between the rates of STAS detection before and after tumor sectioning, in the upper or lower lung parenchyma and in fresh or fixed tissues.^21^ These findings suggest that STAS occurrence is not influenced by gross specimen handling procedures, indicating that it likely represents a biological phenomenon inherent to the tumor rather than an artifact introduced during processing of the surgical sample. 

Our study has some limitations. The main limitation was the small size of the patient sample. However, it was a convenience sample, the main objective being to establish a parallel between frozen section examination and histopathological examination, in order to define the former as a valid method for intraoperative diagnosis of STAS. Despite the scarcity of studies on the subject in the literature, we can draw a comparison between out data and what is currently available in the literature. Despite the modest size of our sample, our findings regarding the sensitivity, specificity, PPV, NPV, and accuracy of frozen section examination are comparable to those of previous studies, especially those of Eguchi et al.^17^ The literature seems to converge regarding the specificity of the method, although sensitivity varies across studies, which may be explained by the absence of technical standardization or guidelines imposing standardization of slide analysis. To our knowledge, this is the first prospective analysis of the topic, through which we aim primarily to establish a standardized methodology for the detection of STAS in frozen section pathology that does not rely solely on the experience of the pathologist and can be replicated worldwide, thereby expanding the discussion.

We can conclude that intraoperative frozen section pathology with technical standardization is capable of identifying STAS in patients with lung cancer undergoing resection. Despite the small number of patients evaluated, the PPV, NPV, sensitivity, and specificity demonstrated that the technical standardization applied could be incorporated at other centers and allow advancements that are directly associated with prognosis. In addition, given the high accuracy (92%), it is possible to infer that its inclusion would inform intraoperative decisions regarding the choice between lobectomy and sublobar resection. These preliminary results, in view of the scarcity of studies on the subject, bring the topic to the cutting edge of the debate on sublobar resection, given the impact on tumor recurrence in STAS-positive patients undergoing resection smaller than lobectomy, that impact being even more pronounced in patients with good pulmonary reserve.
